# Variations in the structure of airborne bacterial communities in Tsogt-Ovoo of Gobi desert area during dust events

**DOI:** 10.1007/s11869-016-0430-3

**Published:** 2016-08-26

**Authors:** Teruya Maki, Yasunori Kurosaki, Kazunari Onishi, Kevin C. Lee, Stephen B. Pointing, Dulam Jugder, Norikazu Yamanaka, Hiroshi Hasegawa, Masato Shinoda

**Affiliations:** 1 College of Science and Engineering, Kanazawa University, Kakumamachi, Kanazawa, Ishikawa 920-1192 Japan; 2 Arid Land Research Center, Tottori University, 1390 Hamasaka, Tottori, 680-0001 Japan; 3 Interdisciplinary Graduate School of Medicine, University of Yamanashi, 1110, Shimokato, Chuo, Yamanashi, 4093898 Japan; 4 School of Applied Sciences, Auckland University of Technology, Private Bag 92006, Auckland, 1142 New Zealand; 5 Information and Research Institute of Meteorology, Hydrology and Environment, Juulchny gudamj-5, Ulaanbaatar-46, 14201 Mongolia; 6 Graduate School of Environmental Studies, Nagoya University, Furocho, Chikusaku, Nagoya, 464-8601 Japan

**Keywords:** Dryland, Bioaerosol, Prokaryote, MiSeq sequencing, Fluorescence microscopy

## Abstract

Asian dust events transport the airborne bacteria in Chinese desert regions as well as mineral particles and influence downwind area varying biological ecosystems and climate changes. However, the airborne bacterial dynamics were rarely investigated in the Gobi desert area, where dust events are highly frequent. In this study, air samplings were sequentially performed at a 2-m high above the ground at the sampling site located in desert area (Tsogt-Ovoo of Gobi desert; Mongolia 44.2304°N, 105.1700°E). During the dust event days, the bacterial cells and mineral particles increased to more than tenfold of concentrations. MiSeq sequencing targeting 16S ribosomal DNA revealed that the airborne bacteria in desert area mainly belonged to the classes *Acidobacteria*, *Actinobacteria*, *Bacteroidetes*, *Chloroflexi*, *Bacilli*, *Alpha-proteobacteria*, *Beta-proteobacteria*, and *Gamma-proteobacteria.* The bacterial community structures were different between dust events and non-dust events. The air samples collected at the dust events indicated high abundance rates of *Alpha-proteobacteria*, which were reported to dominate on the leaf surfaces of plants or in the saline lake environments. After the dust events, the members of *Firmicutes* (*Bacilli*) and *Bacteroidetes*, which are known to form endospore and attach with coarse particles, respectively, increased their relative abundances in the air samples. Presumably, the bacterial compositions and diversities in atmosphere significantly vary during dust events, which carry some particles from grassland (phyllo-sphere), dry lake, and sand surfaces, as well as some bacterial populations such as *Firmicutes* and *Bacteroidetes* maintain in the atmosphere for longer time.

**Electronic supplementary material:**

The online version of this article (doi:10.1007/s11869-016-0430-3) contains supplementary material, which is available to authorized users.

## Introduction

Mineral particles originated from the Chinese desert regions frequently disperse to all around the East Asian regions during the spring season (Iwasaka et al. 1983). The major source areas of the Asian dust event are Gobi desert, Taklimakan desert, and Loess Plateau (Duce et al. 1980; Iwasaka et al. 1983; Kurosaki and Mikami 2005). Desert winds from the Gobi desert area carry several billion tons of soil-derived dust each year (Uematsu et al. 1983; Duce et al. 1980; Chung and Kim 2008; Kim and Chung 2010; Huang et al. 2010), which negatively impacts human health (Onishi et al. 2012) and downwind ecosystems (Pointing and Jayne 2014). In contrast, Asian-dust depositions have some positive effects on ecosystems for moderating acid rain and providing the nutrients to oligotrophic oceans (Pointing and Jayne 2014). Microorganisms (including viruses, bacteria, and fungi) associated with mineral-dust particles, known as “bioaerosols” (Prospero et al. 2005; Kellogg and Griffin 2006; Iwasaka et al. 2009), are also transported over long distances. The dust event dispersal of bioaerosol is linked to the increase of the allergen burden and asthma (Ichinose et al. 2005; Liu et al. 2014) and possibly the dispersal of diseases such as Kawasaki disease in humans (Rodó et al. 2011) and rust diseases in plants (Brown and Hovmøller 2002). Moreover, bioaerosols are thought to influence atmospheric processes by participating in atmospheric chemical reactions and cloud particle formation (Pratt et al. 2009; Creamean et al. 2013).

To understand the long-range transport processes of bioaerosols, the dynamics of airborne prokaryotic communities over the Asian-dust source regions should be investigated. The airborne bacteria were known to be mixed vertically at high altitudes above the oasis city, Dunhuang, in Taklimakan desert (Maki et al. 2008; Kakikawa et al. 2008). The desert sand included several kinds of bacterial species, and some of the bacterial population would be transported to atmosphere over ground surfaces (An et al. 2013; Puspitasari et al. 2015). While the airborne bacteria around the Mogao Caves in Dunhuang were also investigated, the bacterial variations depended on the numbers of tourists visiting the caves (Wang et al. 2010). Therefore, in order to study the natural origins of airborne bacteria in desert, the sampling site should be located in sandy desert area, where dust events occurred frequently and human activities can be avoided. Dust storms in the Gobi desert are more severe and occur more frequently than the storms in Taklimakan desert (Jugder et al. 2004; Kurosaki and Mikami 2005). However, there are few researches investigating the dynamics of airborne bacteria at the ground level in the Gobi desert area.

Kurosaki and Mikami (2007) analyzed East Asian meteorological observatory data and suggested that the highest frequency of dust storms in the region occurred at Tsogt-Ovoo in the middle of the Gobi desert. Located in a shallow valley, Tsogt-Ovoo exemplify topographical depressions known for significant sources of dust originated from dry lakes (Abulaiti et al. 2014). We sequentially collected bioaerosol samples at Tsogt-Ovoo during the dust events in the spring season of 2014 and 2015 to investigate the change in airborne prokaryotic communities. The variations of prokaryotic abundances and compositions in the samples were estimated using microscopic observation and high-throughput sequencing techniques.

## Materials and methods

### Sampling of aerosol and sand particles

**Fig. 1:**
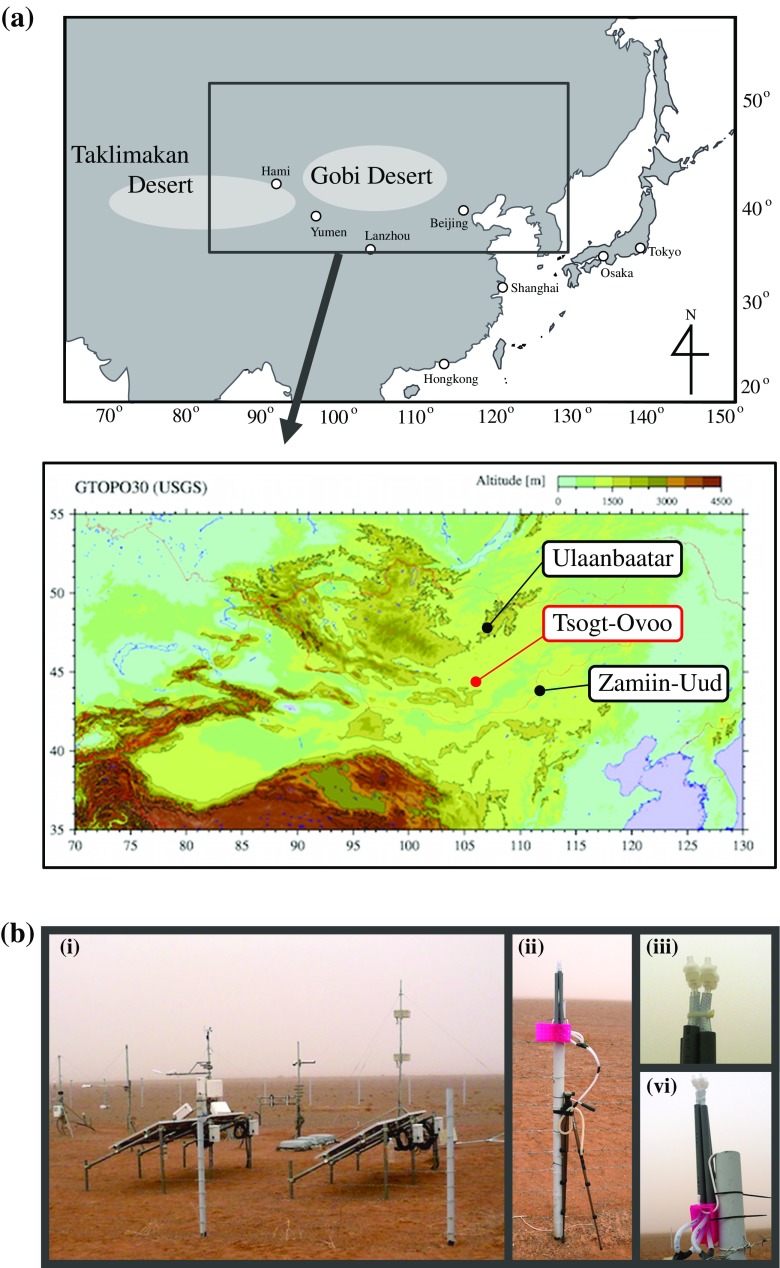
**a** Sampling site of Tsogt-Ovoo City in Asian-dust source regions (Gobi desert) and **b** (i) metrological monitoring systems and (ii–vi) bioaerosol sampling situations

Air samples were collected in Tsogt-Ovoo (Fig. 1) in Mongolia from 16 to 18 March 2014, from 7 to 11 March 2015, and from 26 to 27 April 2015. The sampling times are indicted in Table 1. Tsogt-Ovoo is located in the middle of the Gobi desert, which is a major source of dust traveling toward Japan (Fig. 1a). The sampling site (44.2304°N, 105.1700°E) was located at the desert area 5 km from downtown, and the sampling system was placed on a 2-m-high bar, which was fixed with fences (Fig. 1b). Air samples were collected using sterilized polycarbonate filters (0.22-μm pore size; Whatman, Tokyo, Japan) with a sterilized filter holder connected to an air pump. For each sample, two filters were used continuously for sampling periods ranging from 10 to 12 h (the sampling air volumes ranged from 180 to 216 L); the filters were changed after each sampling period. In total, 13 air samples were obtained during the sampling periods, which were labeled 14To-1 to 14To-4 and 15To-1 to 15To-9 (Table 1). Of the two filters used to collect each sample, one filter was used to determine the particulate abundances using fluorescence microscopy, and the other was stored at −80 °C before the extraction of genomic DNA for the analysis of prokaryotic community composition.

**Table 1:** Sampling information during the sampling periods

Sample name	Sampling time (ULAT)	Sampling time (UTC)	Total time (h)	Air volume (*L*)	Dust^a^
14To-1	March 16, 2014 10:30–17:00	March 16, 2014 02:30–09:00	6.5	468	Dust
14To-2	March 16, 2014 17:00–29:00	March 16, 2014 09:00–21:00	12	864	Dust
14To-3	March 17, 2014 17:00–29:00	March 17, 2014 09:00–21:00	12	864	Non-dust
14To-4	March 18, 2014 11:00–16:00	March 18, 2014 03:00–8:00	5	360	Non-dust
15To-1	March 7, 2015 18:00–34:00	March 7, 2015 10:00–26:00	16	1152	Dust
15To-2	March 8, 2015 10:00–22:00	March 8, 2015 2:00–14:00	12	864	Non-dust
15To-3	March 9, 2015 10:00–18:30	March 9, 2015 02:00–10:30	8.5	612	Non-dust
15To-4	March 9, 2015 19:00–33:30	March 9, 2015 11:00–25:30	14.5	1008	Non-dust
15To-5	March 10, 2015 10:30–17:30	March 10, 2015 02:30–9:30	7	504	Non-dust
15To-6	March 10, 2015 18:00–33:00	March 10, 2015 10:00–25:00	15	1080	Non-dust
15To-7	March 11, 2015 09:30–16:00	March 11, 2015 01:30–8:00	6.5	468	Non-dust
15To-8	April 26, 2015 08:00–18:30	April 26, 2015 00:00–10:30	10.5	756	Dust
15To-9	April 27, 2015 08:00–18:30	April 27, 2015 00:00–10:30	10.5	792	Non-dust

^a^The occurrences of dust events are evaluated by depending on lidar data, PM^10^ concentrations, or wind speeds

### Characteristics of atmospheric conditions

The occurrences of dust events were evaluated by measuring concentrations of particulate matter with a median aerodynamic diameter of 10 μm (PM^10^), which were measured at a height of 1.42 m using an aerosol mass monitor (DustTrak^™^ DRX 8533, TSI Inc., Shoreview, MN, USA). Wind speed and wind direction were determined at a height of 3 m using a propeller anemometer (YG-5103, R.M. Young, Traverse City, MI, USA). The dust concentration was measured only when the wind speed exceeded 8 m/s. All data were automatically measured every 0.1 s for wind speed and every second for PM^10^ and wind direction. The averaged measurement values over 1-min intervals were recorded to the data loggers (CR1000-XT, Campbell Scientific Inc., North Logan, UT, USA). These observation systems have been established for monitoring dust conditions and meteorological factors (Ishizuka et al. 2012).

The depolarization rates measured by the lidar system of Zamiin-Uud were also used for evaluating dust-event occurrences over the Gobi desert area.

### Microscopic analysis of particle abundance

To determine the particle abundance, 0.25 mL of sterilized ultrapure water with paraformaldehyde at a final concentration of 1 % was added to one of the filter folders to fix the aerosols (Maki et al. 2014). After a 1-h incubation, the filter was stained with 4,6-diamidino-2-phenylindole (DAPI) at a final concentration of 0.5 μg/mL for 15 min (Porter and Feig 1980). Particles on the filter were observed using an epifluorescence microscope (Olympus, Tokyo, Japan) equipped with an ultraviolet excitation system. A filter transect was scanned, and the mineral particles (white particles), yellow particles, and bacterial cells on the filter transect were counted. Yellow particles stained with DAPI were reported as organic matter (Mostajir et al. 1995). The detection limit of aerosols was below 5 × 10^3^ particles/m^3^ of air.

### High-throughput sequencing

After the sampling, the aerosols were washed off the filters by shaking with 5 mL of sterilized water containing 0.9 % (*w*/*v*) of NaCl, and the solution samples were pelleted by centrifugation at 20,000*g* for 5 min. Genomic DNA (gDNA) was extracted using a modified phenol-chloroform method (Maki et al. 2008). Fragments of 16S ribosomal DNA (rDNA; approximately 500 bp) were amplified from the extracted gDNA by PCR using universal 16S rDNA prokaryotic primers for the V4 region, 515F (5′-Seq A-TGT GCC AGC MGC CGC GGT AA-3′) and 806R (5′-Seq B-GGA CTA CHV GGG TWT CTA AT-3′) (Caporaso et al. 2011). This read sequence region provides an accurate taxonomic information at the family level of bacterial composition (Liu et al. 2008). The nucleotide sequences of Seq A and Seq B represent nucleotide sequences targeted by the second set of PCR primers. PCR amplification was performed under the following conditions: denaturation at 94 °C for 1 min, annealing at 52 °C for 2 min, and extension at 72 °C for 2 min for 20 cycles. Fragments of 16S rDNA in PCR products were amplified again using the second PCR forward primers (5′-adaptor C—tag sequence Seq A-3′) and reverse primer (5′-adaptor D—Seq B-3′), where adaptors C and D were used for the MiSeq sequencing reaction. The tag sequence included eight nucleotides designed for sample identification bar coding. Thermal cycling was performed under the following conditions: denaturation at 94 °C for 1 min, annealing at 59 °C for 2 min, and extension at 72 °C for 2 min for 14 cycles. PCR amplicons from each sample were used for high-throughput sequencing on a MiSeq Genome Sequencer (Illumina, CA, USA). The paired-end sequences obtained, with the read length of 250 bp, were grouped based on tag sequences for each sample. Negative controls (no template and template from unused filters) were prepared in all steps of the process after DNA extraction to check for contamination.

Before the analysis of bacterial community structures, USEARCH v.8.01623 (Edgar 2013) was used to process the raw Illumina sequencing reads. Anomalous sequences were removed with the following workflow. First, the forward and reverse paired-end reads were merged, and the merged reads with lengths outside 200–500-bp range or exceeding six homopolymers were discarded by Mothur v1.36.1 (Schloss et al. 2009). Next, the sequences were subjected to Q-score filtering to remove reads with more than one expected error. Reads occurring only once in the entire dataset (singleton) were then removed. Theses sequences were clustered de novo (with a minimum identity of 97 %) into 1065 operational taxonomic units (OTUs) among the 13 samples. The taxonomies of the representative OTU sequences were assigned using the RDP classifier (Wang et al. 2007) implemented in QIME v9.1.1 (Caporaso et al. 2010). Greengenes release 13_8 (McDonald et al. 2012) was used as the reference taxonomic database. All sequences have been deposited in the DDBJ database (accession number of the submission is DRA005058).

## Results and discussion

**Fig. 2:**
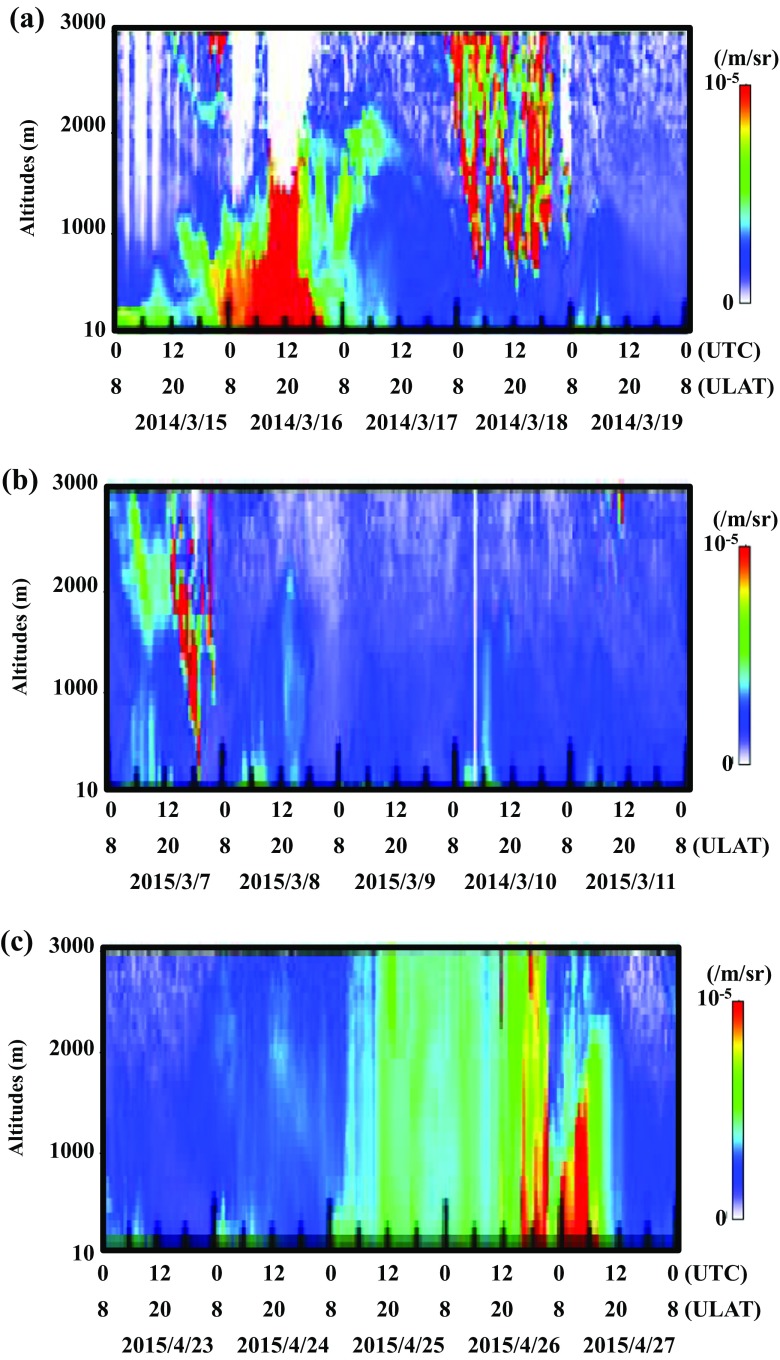
Lidar observation of depolarization ratio in Zamiin-Uud from 00:00 UTC on March 15 to 00:00 UTC on March 19 in 2014 (**a**), from 00:00 UTC on March 7 to 00:00 UTC on March 11 in 2015 (**b**), and from 00:00 UTC on April 23 to 00:00 UTC on April 27 in 2015 (**c**)

**Fig. 3:**
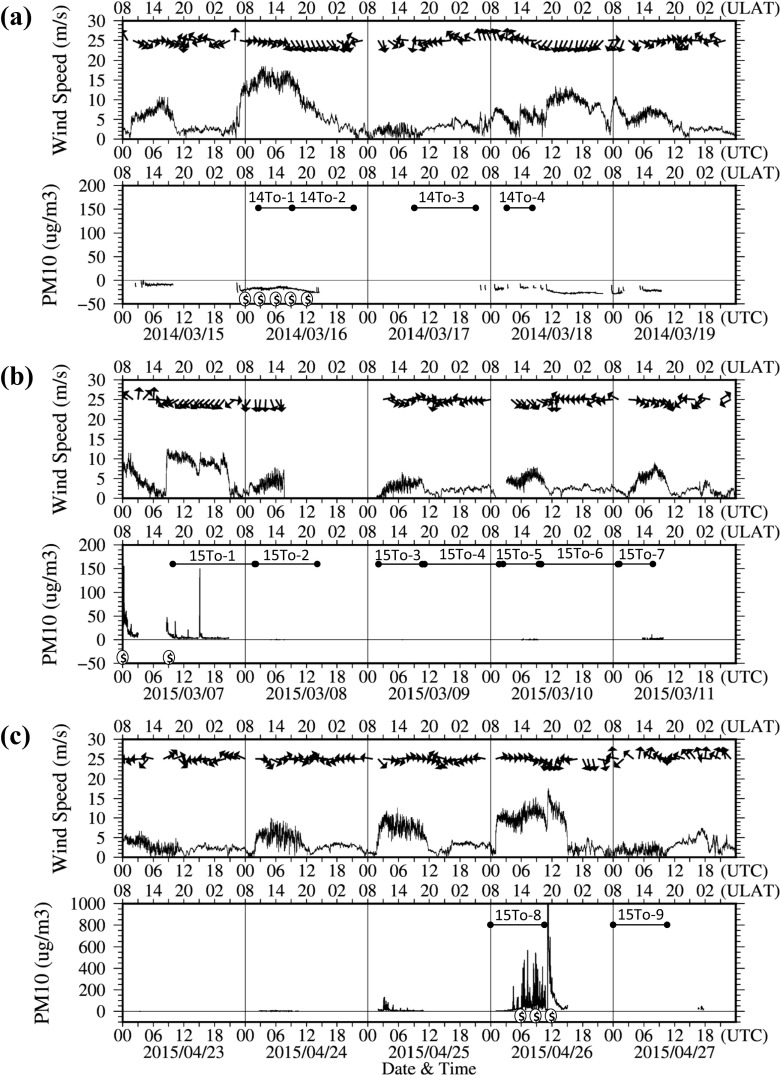
Wind speed and PM^10^ concentrations in the atmosphere at the sampling site of Tsogt-Ovoo City from 00:00 UTC on March 15 to 00:00 UTC on March 19 in 2014 (**a**), from 00:00 UTC on March 7 to 00:00 UTC on March 11 in 2015 (**b**), and from 00:00 UTC on April 23 to 00:00 UTC on April 27 in 2015 (**c**). There are no data between 14:30 on March 8 and 11:00 on March 9 in 2015. The optical-particle counter had errors on March in 2014 and indicated minus values of PM^10^ concentrations. The *dollar sign* means the occurrences of dust events, which are shown on the weather charts of Tsogt-Ovoo City

The depolarization rates increased between low and high altitudes on March 16, 2014; March 7 and 8, 2015; and April 26 and 27, 2015 (Fig. 2), suggesting the occurrence of dust events over the Gobi desert during those time periods. In particular, the dust event of March 16, 2014 could be observed on the satellite-monitoring chart of MODIS sensor (Fig. S1). During the sampling periods on March 2015 and April 2015, the concentrations of PM^10^ significantly increased under strong wind exceeding 10 m/s, indicating the occurrences of dust events on March 7, 2015 and April 26, 2015 (Fig. 3). Unfortunately, the aerosol mass monitor indicated minus values on March 2014 due to the mismatch of calculation software with device, so particulate concentration of the strong dust event on March 16, 2014 could not be used (Fig. 3a). In the Gobi desert area, wind speed is an important factor for the saltation of mineral particles from the ground surfaces (Ishizuka et al. 2012) and the occurrences of dust events (Kurosaki and Mikami 2005). The wind directions generally change at ground level in the Gobi desert, while the westerly wind is blowing at the high altitudes of more than 1000 m constantly and carry the mineral particles to the East Asia regions for long distance (Iwasaka et al. 1983). Consequently, we classified the samples of 14To-1, 14To-2, 15To-1, and 15To-8 as air samples including the dust particles transported by dust events (Table 1). The weather charts of Tsogt-Ovoo also support the occurrences of dust event on March 16, 2014; March 7, 2015; and April 26, 2015. Accordingly, the samples of 14To-1, 14To-2, 15To-1, and 15To-8 were named as “dust samples,” and the samples of 14To-3, 14To-4, 15To-2, 15To-3, 15To-4, 15To-5, 15To-6, 15To-8, and 15To-9 were named as “non-dust samples.”

**Fig. 4:**
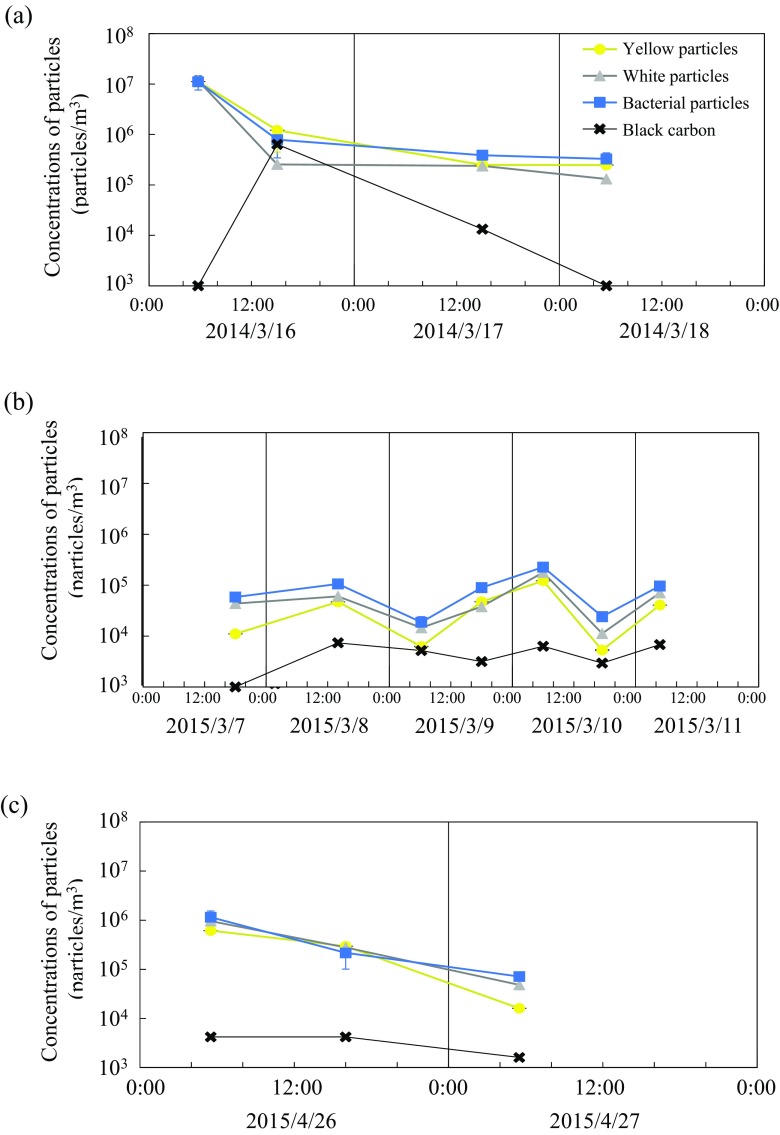
Changes in fluorescent particle concentrations at a 2-m high above the ground at the sampling site of Tsogt-Ovoo City for March 16–18 in 2014 (UTC) (**a**), March 7–11 in 2015 (UTC) (**b**), and April 26–27 in 2015 (UTC) (**c**). The *error bars* were obtained from the count numbers obtained from the ten fields of microscopic observation

Under microscopic observation using DAPI staining, some fluorescent particles were observed in the subsamples, and they were mainly composed of bright-blue fluorescence particles (prokaryotic cells), white-blue fluorescent particle (mineral particles), and yellow fluorescent particles (organic matters). During non-dust event periods, low concentrations of particles, in the orders of 10^4^ to 10^5^ particles/m^3^, were observed. The total particle concentrations of dust samples 14To-1, 14To-2, and 15To-8 increased to more than 10^6^ particles/m^3^ and sometimes reaching 10^7^ particles/m^3^ (Fig. 4). When dust events occurred, the airborne prokaryote maintained high concentration ranging from 10^6^ to 10^7^ particles/m^3^. The airborne bacteria in downwind environments in East Asia are reported to maintained lower cell concentrations ranging from 10^4^ to 10^6^ particles/m^3^ (Hara and Zhang 2012; Maki et al. 2014). In the dust source region, dust events would often occur increasing airborne microbial abundances, which are at least one order higher than downwind environments.

**Fig. 5:**
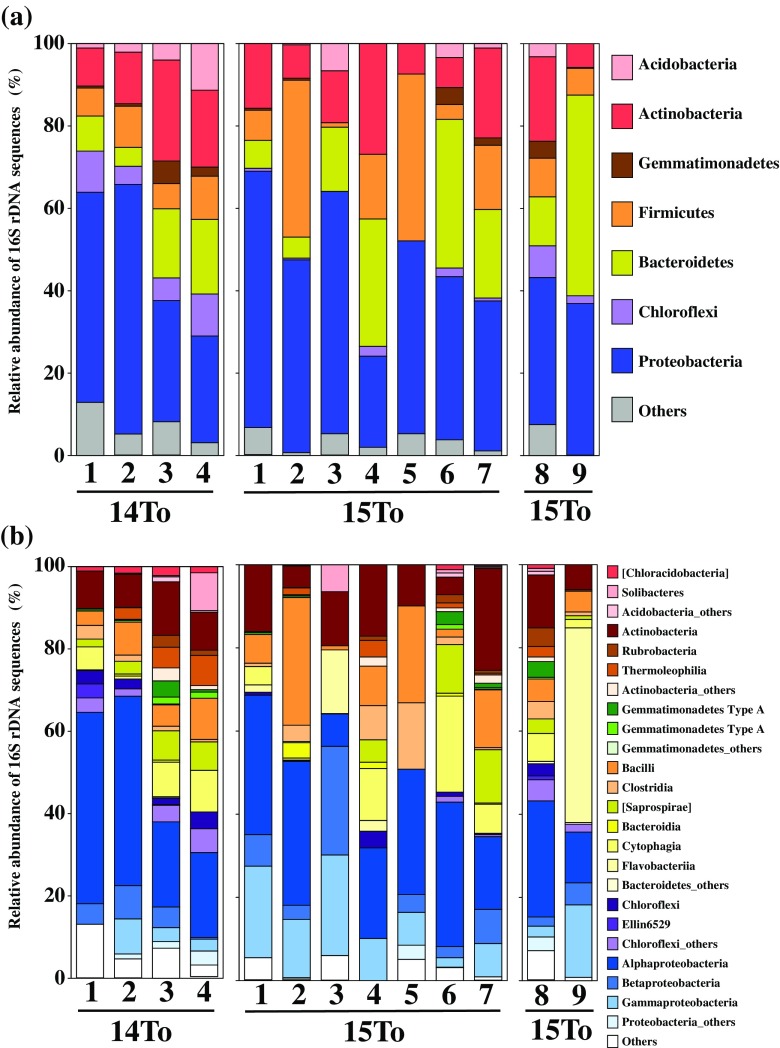
Bacterial composition at the phylum (**a**) and class (**b**) levels of the partial sequences in the MiSeq sequencing database obtained from the air samples collected at the sampling site of Tsogt-Ovoo City for March 16–18 in 2014 (from 14To-1 to 14To-4), March 7–11 in 2015 (from 15To-1 to 14To-7), and April 26–27 in 2015 (15To-8 and 15To-9)

**Fig. 6:**
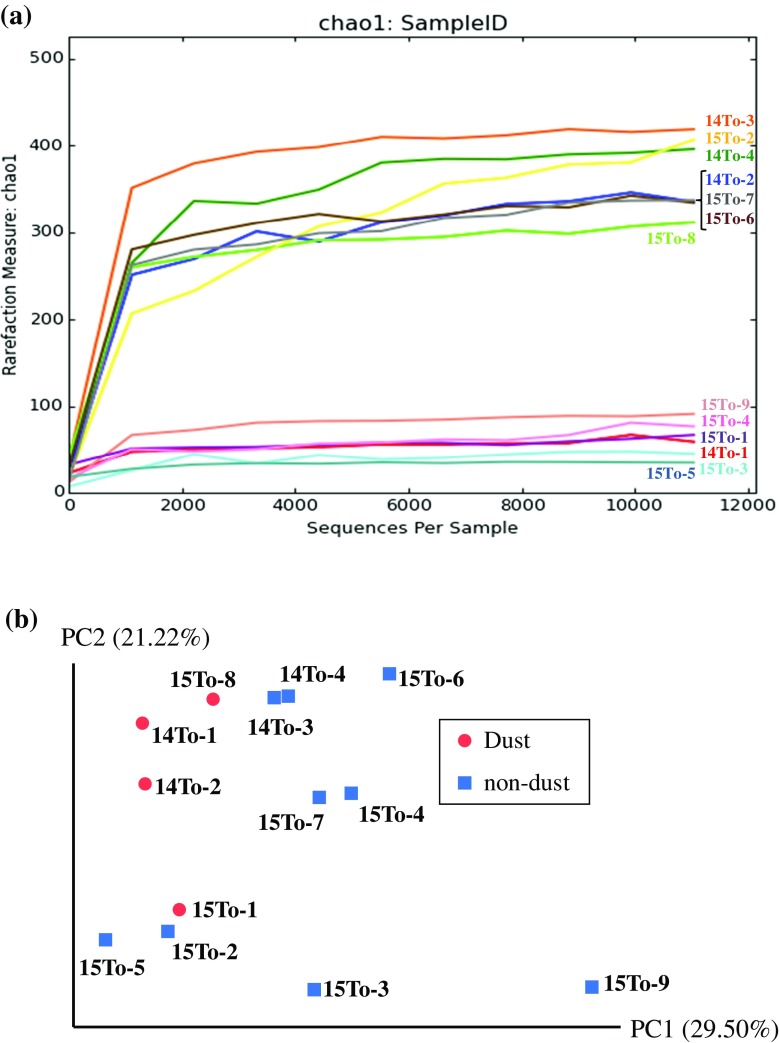
Comparison of bacterial compositions among the air samples collected at the sampling site of Tsogt-Ovoo City for March 16–18 in 2014 (from 14To-1 to 14To-4), March 7–11 in 2015 (from 15To-1 to 14To-7), and April 26–27 in 2015 (15To-8 and 15To-9). **a** Rarefaction curves indicating the bacterial diversity observed in the air samples. Species were binned at the 97 % sequence similarity level. **b** Non-metric multidimensional scaling plot with weighted-UniFrac distance matrix displaying phylogenetic clustering by the air samples

For the analysis of prokaryotic compositions in the air samples, we obtained a total of 2,503,780 reads. Following quality filtering, 1,113,730 merged paired-end sequences with a median length of 292 bp remained, and sequence library size for each sample was normalized at 9245. The 16S rDNA sequences were divided into 1065 phylotypes (sequences with >97 % similarity). Phylogenetic assignment of sequences resulted in overall diversity comprising 21 phyla and candidate divisions, 66 classes (and class-level candidate taxa), and 196 families (and family-level candidate taxa). The majority (>90 %) of abundance were represented by 7 bacterial phyla and the 24 classes (Fig. 5). The rarefaction curves among the 13 samples could be distinguished to 2 different patterns indicating higher and lower diversities (Fig. 6a). Non-metric multidimensional scaling plot with weighted-UniFrac distances demonstrated the distinct clustering of prokaryotic communities separating dust samples and non-dust samples (Fig. 6b). In general, among environmental prokaryotic communities, airborne prokaryotes showed the lowest diversities, whereas the terrestrial bacteria did the highest diversities (Fierer and Lennon 2011). Archaea in two phyla (*Thaumarchaeota* and *Euryarchaeota*) were detected but were in relatively low abundance compared to the dominant bacteria phyla. The bacterial compositions varied during the sampling periods and were mainly composed of the phylotypes belonging to the phyla *Actinobacteria*, *Firmicutes*, *Bacteroidetes*, and particularly *Proteobacteria*. These bacterial members are typically generated from atmospheric and terrestrial environments in the Gobi and Taklimakan deserts (Fig. 5a; An et al. 2013; Puspitasari et al. 2015). Dust events in desert areas may have provided terrestrial particles to the atmosphere and thus increased the diversities of airborne microbial communities. After such dust events, some of the microbial species that were resistant to atmospheric stressors would remain as dominated members of airborne microbiome.

The sequences of the class *Alpha-proteobacteria* showed relatively high abundances ranging from 28 to 46 % in the samples collected during dust events, while they decreased to no more than 20 % in the non-dust samples except for samples 15To-5 and 15To-6 (Fig. 5b). The several members of *Alpha-proteobacteria* were known to account for bacterial population associated with plant body or surfaces (Fürnkranz et al. 2008). Some desert grasses randomly distribute around the ground surfaces in the Gobi desert regions (Shinoda et al. 2010; Jamsran et al. 2015). The *Alpha-proteobacteria* members predominately occupy the bacterial communities in high-saline environments such as marine and salt lakes (Desriac et al. 2013). In arid areas, minerals including sodium chloride are accumulated on sand-ground surfaces, causing saline conditions in desert area (Dianwu et al. 1988). The sampling site in Tsogt-Ovoo is located in a shallow valley, where some rainwater is completely desiccated forming dry lakes with high-saline conditions (Engelstaedter et al. 2003). The phyllo-sphere of desert grasses and the dry-lake environments may contribute to the airborne bacteria from ground surfaces. After dust events, weak winds hardly transport the bacterial population from desert-grass surfaces and dry-lake environments, and *Alpha-proteobacteria* members could not maintain their viabilities in the atmosphere, where environmental stressors such as temperature changes, UV irradiances, and extreme desiccation damaged bacterial cells.

During some sampling periods after dust occurrences, phylotypes of the phyla *Firmicutes* and *Bacteroidetes* increased to high relative abundances (Fig. 4). The abundances of *Firmicutes* sequences in the non-dust samples 15To-2, 15To-4, 15To-5, and 15To-7 ranged from 15.7 to 40.5 %, and those of *Bacteroidetes* sequences in the non-dust samples 14To-3, 14To-4, 15To-2, 15To-4, 15To-6, 15To-7, and 15To-9 ranged from 15.6 to 48.7 %, while the abundances of the both phyla in the dust samples 14To-1, 14To-2, 15To-1, and 15To-8 were no more than 12 %. The sequences of *Firmicutes* mainly belong to the classes *Bacilli* and *Clostridia*, which can form resistant endospores enhancing their survival in the atmosphere (Nicholson et al. 2000). Indeed, *Bacilli* sequences were often highly represented in the aerosol samples collected from the Chinese desert and the downwind area during dust events (Jeon et al. 2011; Maki et al. 2015; Puspitasari et al. 2015). The class *Clostridia* includes rumen bacteria dominated in animal guts (Tajima et al. 1999; Lopetuso et al. 2008). Animal fecal pellets are found around the Gobi desert surfaces (Batsaikham et al. 2010), suggesting that they may be a source of airborne bacterial populations. On the other hand, the sequences of *Bacteroidetes* were predominantly composed of the families *Cytophaga* and *Flavobacterium*, which were known to attach onto the organic aggregates in terrestrial and aquatic environments (Turnbaugh et al. 2011). The bacteria attached with coarse particles might maintain their variability more than free bacteria due to the protection of the coarse particles to attached bacteria against atmospheric stressors. In the previous investigation in dust downwind regions such as Japan, members of *Firmicutes* and *Bacteroidetes* were often isolated from the air samples collected during dust events (Hua et al. 2007; Maki et al. 2010; Tanaka et al. 2011; Yamaguchi et al. 2012; Hara et al. 2015). The isolates of *Bacilli* members in *Firmicutes* have been obtained from the air samples collected at an 800-m high above the ground over Dunhuang City in the Taklamakan desert (Maki et al. 2008). Accordingly, these bacteria are thought to keep their viabilities by the resistance to the atmospheric stressors and maintain their populations for longer periods after dust events.

The members of the phyla *Acidobacteria*, *Gemmatimonadetes*, and *Chloroflexi* appeared in regardless of dust events and had the low relative abundances of no more than 10 %. The members of *Acidobacteria* were often obtained from the sequence database recovered from the terrestrial environments of desert area (Smith et al. 2004) and alpine ecosystems (Lipson and Schmidt 2004). Since almost bacterial species belonging to *Acidobacteria* have not been cultured, their ecology and metabolism were unclear (Barns et al. 1999). However, the phylum of *Acidobacteria* is thought to consist of high diversity of sequences that similar is to that of *Proteobacteria*, suggesting their important contribution to terrestrial ecosystems (Barns et al. 1999). Members of the phylum *Gemmatimonadetes*, which were known to adapt to low soil moisture, were often detected from the wide range of arid environments, such as grassland, prairie, alpine soils, and pasture soil (Will et al. 2010). The bacteria of *Chloroflexi* were reported to be abundance bacteria in the soil of grassland (Will et al. 2010) and alpine (Costello and Schmidt 2006). Some particulates associated with bacterial cells would be consistently transported from sand or grassland surfaces around Gobi desert.

## Conclusion

In this field surveys, the dynamics of airborne bacteria and archaea at a dust source region (Gobi desert) were investigated at the sampling site, which is located on the desert dune and far from city area. During non-dust event, the proportions of potentially plant-associated *Alpha-proteobacteria* decreased, while the members of the classes *Firmicutes* and *Bacteroidetes* increased their relative abundances. Dust events would carry the particles attached with plant bodies and the sand particle covered with the crust layers in dry lake, and atmospheric stressors are thought to eliminate the *Proteobacteria* members after dust events. During non-dust events, the members of the classes *Firmicutes* (*Bacilli*) and *Bacteroidetes* are thought to maintain to suspend in the air due to their resistance against the atmospheric stressors. Terrestrial-originated particles may be constantly transported, maintaining the abundance of *Firmicutes* and *Bacteroidetes* members. Clarifying the sources of airborne bacteria from sources, such as terrestrial particles, plant surfaces, and animal fecal, presents an important topic for future studies. In addition, fine particles should be collected separately by distinguishing from bouncing or tumbling particles for analyzing the characterization of airborne bacteria transported for long periods. Further work comparing the microbial communities collected at dust sink regions may help identify species that are transported over long distances by dust events.

## Electronic supplementary material

ESM 1 (PPT 2325 kb)
