# Supplementary material for: Variations in the structure of airborne bacterial communities in Tsogt-Ovoo of Gobi desert area during dust events
Source: Air Qual Atmos Health. 2016 Aug 26;10(3):249–60. doi: 10.1007/s11869-016-0430-3 (PMC5348566; doi:10.1007/s11869-016-0430-3)
Supplement: Supplementary file 1 — (PPT 2325 kb) [file 11869_2016_430_MOESM1_ESM.ppt]

## Slide 1
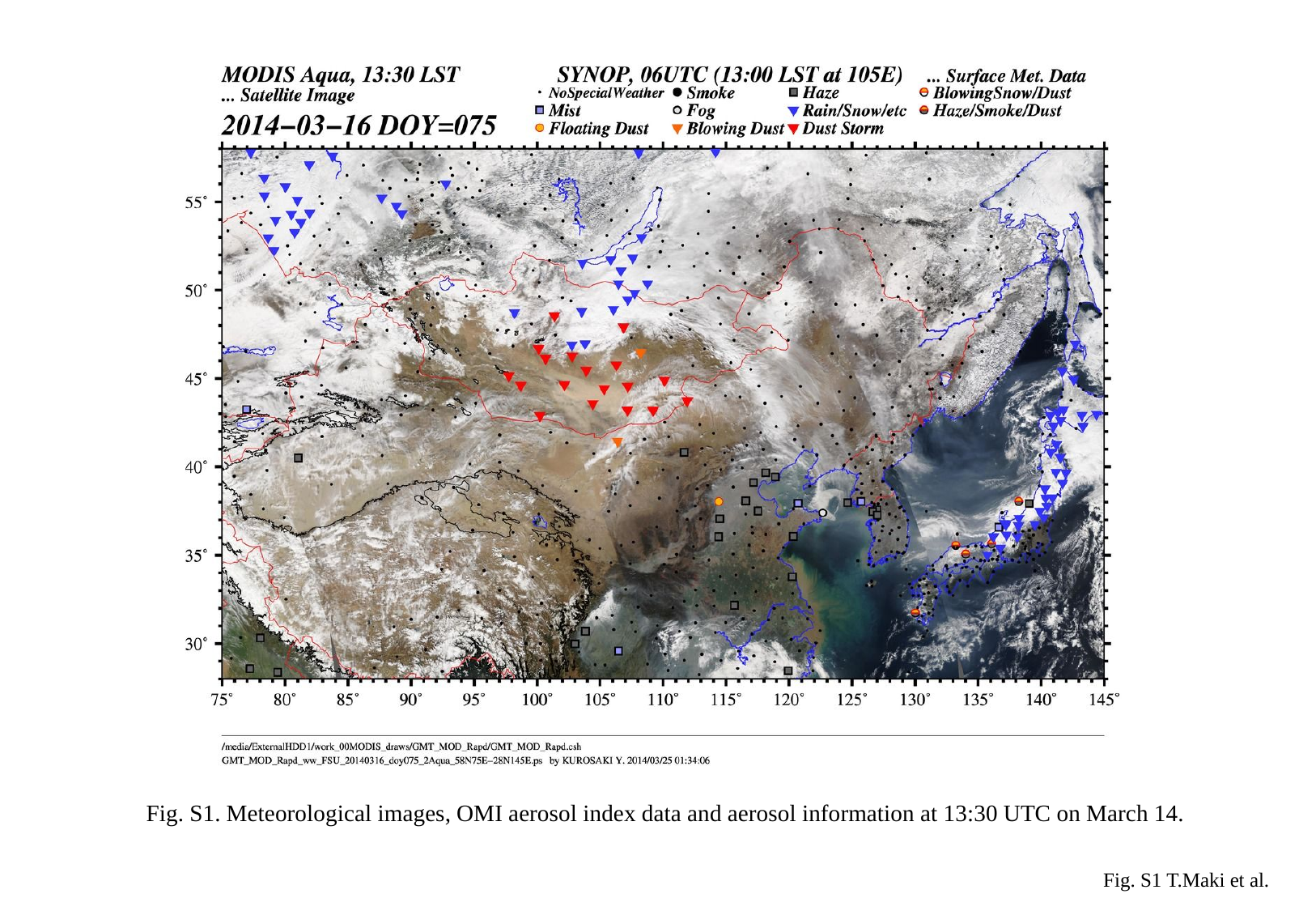

Fig. S1. Meteorological images, OMI aerosol index data and aerosol information at 13:30 UTC on March 14.
Fig. S1 T.Maki et al.
